# Antimicrobial activity of bacteriocin-producing lactic acid bacteria isolated from cheeses and yogurts

**DOI:** 10.1186/2191-0855-2-48

**Published:** 2012-09-10

**Authors:** En Yang, Lihua Fan, Yueming Jiang, Craig Doucette, Sherry Fillmore

**Affiliations:** 1 South China Botanical Garden, Chinese Academy of Sciences, Guangzhou, 510650, China; 2 Agriculture and Agri-Food Canada, Atlantic Food and Horticulture Research Centre, 32 Main Street, Kentville, NS, B4N 1J5, Canada; 3 Kunming University of Science and Technology, Yunnan, China

**Keywords:** Lactic acid bacteria, Bacteriocins, Bacteriocin-like substance (BLS), Antimicrobials, Fresh-cut onions

## Abstract

The biopreservation of foods using bacteriocinogenic lactic acid bacteria (LAB) isolated directly from foods is an innovative approach. The objectives of this study were to isolate and identify bacteriocinogenic LAB from various cheeses and yogurts and evaluate their antimicrobial effects on selected spoilage and pathogenic microorganisms *in vitro* as well as on a food commodity.

LAB were isolated using MRS and M17 media. The agar diffusion bioassay was used to screen for bacteriocin or bacteriocin-like substances (BLS) producing LAB using *Lactobacillus sakei* and *Listeria innocua* as indicator organisms. Out of 138 LAB isolates, 28 were found to inhibit these bacteria and were identified as strains of *Enterococcus faecium, Streptococcus thermophilu*s, *Lactobacillus casei* and *Lactobacillus sakei* subsp*. sakei* using 16S rRNA gene sequencing. Eight isolates were tested for antimicrobial activity at 5°C and 20°C against *L. innocua, Escherichia coli*, *Bacillus cereus*, *Pseudomonas fluorescens*, *Erwinia carotovora,* and *Leuconostoc mesenteroides subsp. mesenteroides* using the agar diffusion bioassay, and also against *Penicillium expansum*, *Botrytis cinerea* and *Monilinia frucitcola* using the microdilution plate method. The effect of selected LAB strains on *L. innocua* inoculated onto fresh-cut onions was also investigated.

Twenty percent of our isolates produced BLS inhibiting the growth of *L. innocua* and/or *Lact. sakei.* Organic acids and/or H_2_O_2_ produced by LAB and not the BLS had strong antimicrobial effects on all microorganisms tested with the exception of *E. coli*. *Ent. faecium*, *Strep. thermophilus* and *Lact. casei* effectively inhibited the growth of natural microflora and *L. innocua* inoculated onto fresh-cut onions. Bacteriocinogenic LAB present in cheeses and yogurts may have potential to be used as biopreservatives in foods.

## Introduction

Lactic acid bacteria (LAB) are generally recognized as safe (GRAS microorganisms) and play an important role in food and feed fermentation and preservation either as the natural microflora or as starter cultures added under controlled conditions. The preservative effect exerted by LAB is mainly due to the production of organic acids (such as lactic acid) which result in lowered pHs ([Daeschel 1989]). LAB also produce antimicrobial compounds including hydrogen peroxide, CO_2_, diacetyl, acetaldehyde, D-isomers of amino acids, reuterin and bacteriocins (Cintas et al. [2001]).

Bacteriocins are ribosomally synthesized antimicrobial peptides that are active against other bacteria, either of the same species (narrow spectrum), or across genera (broad spectrum) (Bowdish et al. [2005]; Cotter et al. [2005]). Bacteriocins may be produced by both gram negative and gram positive bacteria (Savadogo et al. [2006]). In recent years, bacteriocin producing LAB have attracted significant attention because of their GRAS status and potential use as safe additives for food preservation (Diop et al. [2007]). Nisin, produced by *Lactococcus lactis*, is the most thoroughly studied bacteriocin to date and has been applied as an additive to certain foods worldwide (Delves-Broughton et al. [1996]). Substantial work has been done on the effectiveness of nisin on various spoilage and pathogenic microorganisms such as *L. monocytogenes* and its application in different food products ([Staszewski and Jagus 2008]; Freitas et al. [2008]; Schillinger et al. [2001]). Other bacteriocins such as pediocin, may also have potential applications in foods, though they are not currently approved as antimicrobial food additives (Naghmouchi et al. [2007]).

Fresh fruits and vegetables harbor various microorganisms, some of which are psychrotrophic*. L. monocytogenes* is one of the pathogenic bacteria capable of growing at refrigeration temperatures. Moreover, it is also tolerate to acidic pH and salt concentrations up to 10% ([Tasara and Stephan 2006]; Vescovo et al. [2006]). Therefore, it is important to seek biopreservatives that control both spoilage and pathogenic microorganisms including *L. monocytogenes*. Although several studies have indicated the presence of LAB species with antagonistic activity for improving the quality and safety of meat and dairy products (Gagnaire et al. [2009]; [Stiles and Holzapfel 1997]), few reports have involved fresh produce (Trias et al. [2008a,b]). Since the isolation and screening of microorganisms from natural sources has always been the most powerful means for obtaining useful and genetically stable strains for industrially important products (Ibourahema et al. [2008]), in the prsent study, we isolated and identified bacteriocinogenic LAB from cheeses and yogurts, then further evaluated their antimicrobial effects in *vitro* and on fresh-cut produce inoculated with *L. innocua*, a surrogate bacteria for *L. monocytogenes*.

## Materials and methods

### Isolation and screening of LAB

LAB were isolated from 7 commercial cheeses: [Tre Stelle® bocconcini cheese (TSB); Tre Stelle® fromage romano cheese (TSR); Saputo® feta cheese (SF); Agropur® signature rondoux pure goat cheese (ASR); Agropur® signature OKA cheese (OKA); Arla® fontina cheese (Arla) and Jarlsberg® firm ripened cheese (JFR)]; plus 3 commercially available yogurts: (Danone, Activia®; Astro® BioBest yogurt; and Bioghurt® Liberte yogurt), and one in- house produced yogurt.

A 25 g sample of cheese was weighed into filtered stomacher bags (Fisher Scientific, Nepean, ON, Canada) and mixed with 225 ml of sterile 0.1% (w/v) peptone water (Fisher Scientific). Samples were blended at 280 rpm for 3 min (400C stomacher circulator, Seward, England). For yogurt samples, 1 ml of sample was added to 99 ml of sterile 0.1% peptone water. All samples were serially diluted and 50uL of each dilution was spiral plated onto de Man, Rogosa and Sharp (MRS) agar, (Oxoid, Basingstoke, UK) and M17 agar (Oxoid). MRS plates were incubated at 37°C under both aerobic and anaerobic conditions for 48 h and M17 plates at 44°C under anaerobic condition for 48 h. All gram positive, catalase negative (3% v/v H_2_O_2_) isolates were purified and observed under a light microscope. All isolates were coded and stored in MRS or M17 broth containing equal amounts of 30% sterile glycerol at - 80°C.

### Antimicrobial activity and enzymatic testing of bacteriocin producing LAB

The agar diffusion bioassay described by Herreros et al. ([2005]) was used to screen for bacteriocin producing LAB among the 138 isolates. *L. innocua* (ATCC 33090^TM^) and *Lact. sakei* (ATCC 15521^TM^) were used as indicator bacteria. *L. innocua* was incubated overnight in Brain Heart Infusion broth (BHI, Fisher Scientific, ON, Canada) at 37°C and *Lact. sakei* was cultured anaerobically in MRS broth at 37°C.

One ml of each indicator organism (5 × 10^5^ cfu ml^-1^) was inoculated into 15 ml of semisolid BHI or MRS agar (BHI or MRS broth plus 0.7% bacteriological agar) maintained at 50°C and then poured into a petri dish. After solidification, three wells (5 mm diameter) were cut and 35 μl of cell-free supernatant (CFS) from each LAB isolate and appropriately adjusted was added to each well. CFS were prepared as follows: one ml of frozen LAB isolate was cultured overnight in 20 ml MRS or M17 broth then 1 ml culture was sub-cultured overnight in 20 ml MRS broth. Cells were removed by centrifuging at 14,000 g for 5 min (Sorvall RC6 PLUS, Thermo-electron Corporation, Asheville, NC, USA). The supernatant was filtered through a sterile 0.22 μm syringe filter (Chromatographic Specialties Inc., ON, Canada) and 35 μl of the unadjusted aliquot of CFS was added to the first well. The remaining CFS was adjusted to pH 6.0 with 1 mol l^-1^ NaOH in order to rule out possible inhibition effects due to organic acids. 35 μl of the pH adjusted CFS was filtered and added to the second well. The neutralized CFS was then treated with 1 mg ml^-1^ of catalase (Sigma-Aldrich Corporation, USA) at 25°C for 30 min to eliminate the possible inhibitory action of H_2_O_2_ and filtered, then was placed in the third well. If inhibitions zones were found in the third well, the isolates were considered to be able to produce BLS.

The BHI or MRS plates were incubated at 37°C aerobically for 24 h or at 37°C anaerobically for 24 h, respectively. Inhibition zones were measured using an electronic caliper with digital display (Mastercraft^MD^, Miami, FL, USA). Screenings for bacteriocin producing LAB were repeated twice for each isolate.

To confirm production of a proteinaceous compound, CFS displaying antimicrobial potential after acid neutralization and H_2_O_2_ elimination were treated with 1 mg ml^-1^ of proteolytic enzymes, including proteinase K (33 U mg^-1^), α-chymotripsin (66 U mg^-1^), and trypsin (105 U mg^-1^) (Sigma- Aldrich Corporation, USA) at 37°C for 2 h (Bonadè et al. [2001], Herreros et al. [2005]). Antimicrobial activity of treated CFS was determined by the agar diffusion bioassay as described above.

### Identification of BLS producing LAB using 16S rRNA gene sequencing

Near full-length 16S rRNA gene sequencing was used to identify unknown bacteriocin producing LAB strains based on the method of Abnous et al. ([2009]). Briefly, genomic DNA was extracted from isolates using the UltraClean^TM^ Microbial DNA Isolation kit (MO Bio laboratories, Inc., Carlsbas, CA, USA). Universal primers F44 (5’RGTTYGATYMTGGCTCAG-3’) and R1543 (5’-GNNTACCTTKTTACG ACTT-3’) (Abnous et al., [2009]) were used for the amplification of the 16S rRNA gene by PCR. PCR reactions were carried out using a Biometra thermal cycler (Montreal Biotech Inc., Kirkland, QC, Canada) with the following cycle parameters: an initial denaturation at 94°C for 2 min, followed by 35 cycles of denaturation at 94°C for 30 s, annealing at 52°C for 30 s, and elongation at 72°C for 1 min. A final elongation step was performed at 72°C for 5 min. PCR amplicons were separated by agarose gel electrophoresis (0.8% w/v) and visualized by staining with ethidium bromide.

The PCR products were cloned into the psc-A-amp/kan vector using the StrataClone PCR Cloning kit (Stratagene, La Jolla, CA) and transformed into *E. coli* according to the manufacturer’s instructions. Transformants were grown overnight in Luria-Bertani broth supplemented with 100 μg ml^-1^ ampicillin. Plasmids were extracted and purified from selected *E. coli* clones with the UltraClean ^TM^ mini plasmid prep kit (MO Bio Laboratories) according to the manufacturer’s recommendations and then sequenced using the Big Dye Terminator v3.1 cycle sequencing kit. A homology search of the sequences was conducted using the BLAST program at the NCBI database.

### Thermal stability of bacteriocins produced by LAB isolates

Based on identified LAB species, isolation source and the size of inhibition zones, eight LAB isolates were chosen for thermal stability tests. The pH adjusted and H_2_O_2_ eliminated CFS described above were treated at 80 and 100°C for both 60 and 90 min, and at 121°C for 15 min. pH adjusted and H_2_O_2_ eliminated CFS without any heat treatments served as a controls. Residual antimicrobial activity of heat-treated CFS was determined by the agar diffusion bioassay compared to the control using *L. innocua* as the indicator bacteria.

### Antibacterial effects of LAB isolates *in vitro*

The antibacterial effects of eight selected LAB isolates on six common food borne pathogens or spoilage organisms at 5 and 20°C were investigated using the agar diffusion bioassay described above. Targeted indicator organisms and their respective media used were as follows: *L. innocua* (BHI, Difco^TM^, Spark, MD), *E. coli* K-12 (ATCC 10798^TM^) (Tryptic Soy Broth, Difco^TM^), *B*. *cereus* (ATCC 14579^TM^) (Nutrient Broth (NB), Difco^TM^), *Ps*. *fluorescens* (A7B) (NB), *Erw*. *carotovora* (ATCC 15713^TM^) (NB) and *Leuc*. *mesenteroides* subsp. *mesenteroides* (ATCC 8293^TM^) (MRS, Difco^TM^). All strains were cultured aerobically for 24 h at their optimal growth temperatures: 26°C for *Erw. carotovora, Ps. fluorescens* and *Leuc. mesenteroides*; 30°C for *B. cereus*; and 37°C for *E. coli* and *L. innocua*. LAB isolates were sub-cultured and the CFS was prepared as previously described. Following inoculation, plates were incubated at 5 and 20°C for 7 d and 24 h, respectively. Inhibition zones were measured as before.

### Antifungal effects of LAB isolates *in vitro*

The microdilution method described by Lavermicocca et al. ([2003]) with some modifications was used to test the eight LAB isolates against *P*. *expansum* (Pex 03–10.1), *B*. *cinerea* (B94-b) and *M*. *fructicola* (Mof 03–25) at 5 and 20°C. Fungi were obtained from the culture collection at our Research Centre, AAFC. The stock cultures were stored as spore or mycelial suspensions in 15% glycerol (v/v) at −80°C. Conidia of *P. expansum* were collected from 4 d-old cultures grown on potato dextrose agar (PDA) at 25°C. Conidia of *B. cinerea* were collected from 12 d-old cultures grown under a 12 h light/dark cycle on Pseudomonas Agar F at 22°C. Conidia of *M. fructicola* were collected from 12 d-old cultures grown on modified V-8^TM^ medium at 25°C. Using sterile distilled water, the density of spore suspensions were diluted to 2 × 10^5^ spores ml^-1^ as determined using a haemocytometer (Hausser Scientific, PA, USA).

Microdilution tests were performed in sterile 96-well micro dilution plates (Costar 3370, Corning Incorporated, Corning, NY, USA). 200 μl of test solution consisting of 185 μl LAB CFS inoculated with 15 μl of conidial suspension was dispensed into the wells. Microdilution plates were incubated at either 20 or 5°C and the optical density (OD) at 580 nm was recorded at specific time intervals using a microtiter plate reader/ spectrophotometer (Spectra MAX 190, Molecular Devices, CA, USA). For the 20°C plates, OD values were measured at 0, 24, 40, 48 or 72 h, while at 5°C, they were measured at 0, 48, 72, 96 or 120 h. For each fungus, both positive controls containing 185 μl MRS broth and 15 μl conidial suspension and negative controls containing 185 μl LAB CFS and 15 μl dead conidial suspension were prepared and monitored. Experiments were repeated three times.

### *In vivo* testing of selected LAB on microflora naturally occurring on fresh-cut onions

*Ent. faecium, Strep. thermophilu*s and *Lact. casei* were chosen for *in vivo* tests. Fresh-cut yellow onions processed at a commercial facility were supplied by Nova Agri Inc. (Canning, NS). *Ent. faecium* and *Lact. casei* were incubated anaerobically in MRS broth at 37°C for 16 h. *Strep. thermophilu*s was incubated anaerobically in M17 broth at 43°C for 16 h. LAB were sub-cultured twice and then centrifuged at 14,000 g for 5 min. Pellets of LAB were washed using sterile distilled water, centrifuged, re-suspended and inoculated onto the batches of diced onions using a calibrated TLC sprayer to give a final concentration of 5 × 10^5^ cfu g^-1^. Diced onions inoculated with sterile distilled water served as a control. Samples were left to dry for 10 min, then 100 g was transferred to food grade bags (Golden Eagle-VH-62 190) and sealed using an electric sealer (FoodSaver V2490, Tilia International Inc. US). The densities of naturally occurring (indigenous) LAB, *Pseudomonas* sp., yeasts and moulds, *Listeria* sp*.* and *Enterobacteriaceae* were enumerated following 0, 3, 6, 9 and 12 d of storage at 4°C. LAB were cultured anaerobically on MRS Agar at 37°C for 48 h; *Pseudomonas* sp. on Pseudomonas selective medium at 30°C for 48 h; yeasts and moulds on PDA supplemented with chloramphenicol (Difco^TM^) at 25°C for 48 h; and *Enterobacteriaceae* on Violet Red Bile Agar (VRBG)(Difco^TM^) at 37°C for 48 h. Colonies were counted using an aCOLyte automated colony counter (Synbiosis, Cambridge, England) and expressed as cfu g^-1^ of diced onions.

### *In vivo* testing of selected LAB on *L. innocua* inoculated on fresh-cut onions

*L. innocua* was sub-cultured into BHI broth and incubated at 37°C for 24 h. The selected LAB strains and inoculation procedures were the same as described above. The final density of *L. innocua* inoculated on fresh-cut onions was 5 × 10^3^ cfu g^-1^. After drying for 10 min, the LAB species were inoculated respectively onto the fresh-cut onions at a density of 5 × 10^5^ cfu g^-1^. A batch of fresh-cut onions inoculated with *L. innocua* alone was used as the control. Samples (100 g/ bag) were stored at 4°C and *Listeria* sp*.* and LAB were enumerated following 0, 3, 6, 9 and 12 d. *Listeria* sp*.* was cultured on *Listeria* selective medium at 35°C for 48 h and LAB on MRS agar at 37°C for 48 h anaerobically.

### Statistical analysis

The initial screening for bacteriocin producing LAB from 138 LAB isolates was repeated twice. For antimicrobial tests, a three repetition split-split plot design was used with the eight LAB isolates on the main plot versus the six bacteria (or three fungi) on the sub plot, which was then split into two temperatures (5 and 20°C). For the *in vivo* testing, non-inoculation or inoculation of *L. innocua* was designed on the main plot with the three LAB strains used to investigate the treatment effect on naturally occurring microflora and introduced *L. innocua* on the onions. The sub plot was represented by sample removal at days 0, 3, 6, 9 and 12. Data were analyzed using the ANOVA directive and standard errors of mean (SEM) option of GenStat® (12^th^ Edition, VSN International Ltd, Hemel Hempstead UK, 2009). The results of a Principal Components Analysis (PCA) were discussed in terms of component scores.

## Results

### Isolation of potential LAB and screening for bacteriocin producing LAB

A total of 160 potential LAB strains were isolated from 11 different cheeses and yogurts. Eighty seven isolates were obtained from MRS agar and 73 from M17 medium. Of these, 138 were gram positive and catalase negative, and were cocci or rod in shape. Twenty percent of isolates showed antimicrobial activity against at least one indicator that is presumed to be attributable to BLS, which was determined after the neutralization of pH, and the elimination of H_2_O_2_ from the CFS (Table 1). All 28 LAB isolates had BLS inhibitory effect on *L. innocua*, while BLS produced by 20 isolates were effective against *Lact. sakei*.

**Table 1:** **Inhibition zones (mm) of 28 LAB isolated from commercial cheeses and yogurts using*L. innocua*and*Lact. sakei*as indicator bacteria**

**Isolate**	**GenBank accession number**	***L. innocua***			***Lact. sakei***		
		**Control**^**a**^	**-Acid**^**b**^	**BLS**^**c**^	**Control**	**-Acid**	**BLS**
Arla-1	JX275802	8.65 ± 0.23^d^	7.10 ± 0.15	5.82 ± 0.09	5.12 ± 0.01	5.97 ± 0.01	5.74 ± 0.01
Arla-10	JX275803	8.96 ± 0.30	8.15 ± 0.21	6.47 ± 0.03	6.51 ± 0.18	5.59 ± 0.12	5.50 ± 0.03
Arla-11	JX275804	8.83 ± 0.14	7.71 ± 0.09	6.41 ± 0.06	5.76 ± 0.07	6.90 ± 0.05	6.31 ± 0.10
Arla-12	JX275805	9.26 ± 0.09	8.57 ± 0.10	6.47 ± 0.14	5.82 ± 0.18	6.67 ± 0.10	5.83 ± 0.15
Arla-14	JX275806	9.33 ± 0.05	8.40 ± 0.08	5.87 ± 0.04	5.98 ± 0.17	7.22 ± 0.32	7.41 ± 0.14
Arla-16	JX275807	8.47 ± 0.07	7.84 ± 0.20	5.81 ± 0.17	5.09 ± 0.01	5.98 ± 0.03	5.15 ± 0.01
Arla-17	JX275808	8.52 ± 0.10	7.82 ± 0.12	5.57 ± 0.38	--^e^	--	--
Arla-18	JX275809	9.28 ± 0.08	8.84 ± 0.08	6.09 ± 0.10	6.94 ± 0.08	8.49 ± 0.07	7.95 ± 0.10
ASR-1	JX275810	9.02 ± 0.03	7.88 ± 0.07	5.54 ± 0.03	6.85 ± 0.11	7.68 ± 0.07	6.52 ± 0.07
ASR-5	JX275811	10.02 ± 0.10	8.99 ± 0.18	6.13 ± 0.06	7.16 ± 0.04	6.57 ± 0.03	5.88 ± 0.32
ASR-6	JX275812	10.38 ± 0.07	9.50 ± 0.15	6.59 ± 0.06	8.30 ± 0.10	8.59 ± 0.14	8.06 ± 0.07
ASR-7	JX275813	8.89 ± 0.12	7.21 ± 0.15	5.97 ± 0.01	--	--	--
JFR-1	JX275814	8.62 ± 0.01	7.82 ± 0.16	6.01 ± 0.25	5.10 ± 0.01	5.82 ± 0.01	5.51 ± 0.09
JFR-3	JX275815	8.93 ± 0.27	7.42 ± 0.19	6.34 ± 0.07	--	--	--
JFR-4	JX275816	9.31 ± 0.07	7.74 ± 0.11	5.46 ± 0.04	--	--	--
JFR-5	JX275817	8.22 ± 0.13	7.18 ± 0.05	5.93 ± 0.14	--	--	--
TSB-8	JX275827	10.91 ± 0.15	9.32 ± 0.14	6.28 ± 0.08	10.15 ± 0.11	9.88 ± 0.16	8.90 ± 0.16
OKA-1	JX275818	9.13 ± 0.12	8.18 ± 0.01	6.23 ± 0.01	--	--	--
OKA-3	JX275819	8.47 ± 0.20	7.36 ± 0.31	5.42 ± 0.01	--	--	--
OKA-4	JX275820	8.33 ± 0.03	7.59 ± 0.01	5.96 ± 0.06	--	--	--
OKA-8	JX275821	8.84 ± 0.19	8.24 ± 0.26	5.93 ± 0.07	5.97 ± 0.12	6.14 ± 0.17	5.68 ± 0.09
OKA-9	JX275822	8.60 ± 0.10	7.69 ± 0.13	6.16 ± 0.03	5.30 ± 0.01	6.10 ± 0.07	6.21 ± 0.28
OKA-14	JX275823	10.12 ± 0.20	9.08 ± 0.30	6.03 ± 0.09	8.31 ± 0.09	8.65 ± 0.15	8.01 ± 0.10
OKA-15	JX275824	9.89 ± 0.16	9.60 ± 0.08	6.55 ± 0.08	8.75 ± 0.05	9.15 ± 0.20	8.76 ± 0.13
OKA-19	JX275825	11.24 ± 0.06	9.62 ± 0.10	6.54 ± 0.11	8.78 ± 0.06	9.05 ± 0.22	8.67 ± 0.13
OKA-22	JX275826	9.39 ± 0.04	8.52 ± 0.07	5.92 ± 0.14	7.94 ± 0.15	8.22 ± 0.12	8.43 ± 0.25
Yog-3 L	JX275800	10.42 ± 0.16	9.26 ± 0.03	6.11 ± 0.08	8.36 ± 0.24	8.65 ± 0.12	7.79 ± 0.13
Yog-3S	JX275801	10.65 ± 0.09	9.14 ± 0.10	6.17 ± 0.03	8.23 ± 0.09	8.83 ± 0.13	8.12 ± 0.15

^a^ CFS of LAB isolates without any treatment.

^b^ CFS with pH neutralized to 6.0.

^c^ CFS with pH neutralized to 6.0 and H_2_O_2_ eliminated.

^d^ Means ± standard deviation of two replicate experiments.

^e^ No inhibition detected.

The BLS from all 28 LAB isolates lost their anti-listerial activity following treatment with proteinase K, α-chymotripsin and/or trypsin (Table 2). However, when *Lact. sakei* was used as an indicator, the BLS produced by the different LAB stains had varying activity following treatment with these enzymes. Nevertheless, the lost antimicrobial ability following treatment with proteolytic enzymes indicated the proteinaceous nature of the BLS.

**Table 2:** **The effect of α-chymotripsin, protease and trypsin treatment on inhibitory activity of BLS produced by the 28 LAB isolates against*L. innocua*and*Lact. sakei***

**Isolates**	**control**^**a**^	***L. innocua*(indicator)**			**control**	***Lact. sakei*(indicator)**		
		**Enzyme treatment**				**Enzyme treatment**		
		**α-chymotripsin**	**protease**	**trypsin**		**α-chymotripsin**	**protease**	**trypsin**
Arla-1	+^b^	--^c^	--	--	+	--	--	--
Arla-10	+	--	--	--	+	--	--	--
Arla-11	+	--	--	--	+	--	--	--
Arla-12	+	--	--	--	+	--	--	--
Arla-14	+	--	--	--	+	--	--	--
Arla-16	+	--	--	--	+	--	--	--
Arla-17	+	--	--	--	n.d	n.d	n.d	n.d
Arla-18	+	--	--	--	+	--	--	--
ASR-1	+	--	--	--	+	--	--	--
ASR-5	+	--	--	--	+	--	--	--
ASR-6	+	--	--	--	+	--	+	--
ASR-7	+	--	--	--	n.d	n.d	n.d	n.d
JFR-1	+	--	--	--	+	--	--	--
JFR-3	+	--	--	--	n.d	n.d	n.d	n.d
JFR-4	+	--	--	--	n.d	n.d	n.d	n.d
JFR-5	+	--	--	--	n.d	n.d	n.d	n.d
TSB-8	+	--	--	--	+	--	+	+
OKA-1	+	--	--	--	n.d	n.d	n.d	n.d
OKA-3	+	--	--	--	n.d	n.d	n.d	n.d
OKA-4	+	--	--	--	n.d	n.d	n.d	n.d
OKA-8	+	--	--	--	+	+	--	+
OKA-9	+	--	--	--	+	--	--	--
OKA-14	+	--	--	--	+	--	--	+
OKA-15	+	--	--	--	+	--	+	+
OKA-19	+	--	--	--	+	--	+	+
OKA-22	+	--	--	--	+	--	--	--
Yog-3 L	+	--	--	--	+	--	--	+
Yog-3S	+	--	--	--	+	--	--	+

^a^ Control = pH-neutralized and H_2_O_2_-eliminated CFS without addition of enzymes.

^b^ Inhibition zones present.

^c^ No inhibition zone.

^d^ n.d. = not determined.

### Identification of LAB isolates using 16S rRNA gene sequences

Near-full length sequencing of the 16S rRNA gene for the 28 bacteriocin producing LAB identified the isolates as follows: 24 strains of *Ent. faecium*, two strains of *Strep. thermophilus*, one strain of *Lact. casei* and one strain of *Lact. sakei* subsp*. sakei*.

### Thermal stability of BLS following heat treatments

The BLS produced by the eight selected LAB isolates were heat-treated at 80 and 100°C for 60 and 90 min, respectively. BLS were thermal stable at these heat conditions above as their inhibitory effects against *L. innocua* were retained (Table 3). However, BLS were sensitive to autoclaving at 121°C for 15 min displaying either smaller or no inhibition zones compared to the control. Isolates *Strep. thermophilus* (ASR-1) and *Lact. casei* (JFR-5) totally lost their anti-listerial activity subsequent to exposure to 121°C for 15 min.

**Table 3:** **Thermal stability of BLS produced by the selected LAB following various heat treatments (*L. innocua*was used as an indictor bacterium)**

**Anti-listerial activity of BLS after heat treatment**							
**Isolates**	CFS*	BLS**	**80°C, 60 min**	**80°C, 90 min**	**100°C, 60 min**	**100°C, 90 min**	**121°C, 15 min**
Arla-18	+++^a^	++^b^	++	++	++	++	++
ASR-1	+++	++	++	++	++	+	--^d^
ASR-6	+++	++	++	++	++	++	++
JFR-1	+++	++	++	++	++	++	+
JFR-5	++	+ ^c^	+	+	+	+	--
TSB-8	+++	++	++	++	++	++	+
OKA-14	+++	++	++	++	++	++	+
Yog-3S	+++	++	++	++	++	++	+

^a^ Inhibitory zone >10 mm.

^b^ Inhibitory zone = 7–10 mm.

^c^ Inhibitory zone = 5–7 mm.

^d^ No inhibitory zone.

* CFS: cell free supernatant from LAB culture.

** BLS: CFS following the pH neutralization and H_2_O_2_ elimination.

### Action of LAB isolates against six bacteria *in vitro*

The CFS from the eight LAB isolates significantly inhibited the growth of all bacteria tested (*p* < 0.05) at 5 and 20°C with the exception of *E. coli* (Table 4). Neutralized CFS from isolates Arla-18, ASR-6, JFR-1, OKA-14, and Yog-3S continued to inhibit the growth of *Leuc. mesenterioides* at 5°C. After pH neutralization and H_2_O_2_ elimination, the CFS from the eight LAB isolates had inhibitory effects only on *L. innocua* but not the other test bacteria suggesting that organic acids and /or H_2_O_2_ produced by LAB had strong antimicrobial effects on bacteria tested.

**Table 4:** **Effect of 8 selected LAB isolates on various bacterial species*in vitro***

	***Ps. fluoresecens***		***B. cereus***		***Erw. carotovora***		***Leuc. mesenteroides***		***Leuc. mesenteroides***		***L. innocua***		***L. innocua***		***L. innocua***	
**Isolates**	**(Control**^**a**^**)**		**(Control)**		**(Control)**		**(Control)**		**(−Acid**^**b**^**)**		**(Control)**		**(−Acid)**		**(BLS**^**c**^**)**	
	5°C	20°C	5°C	20°C	5°C	20°C	5°C	20°C	5°C	20°C	5°C	20°C	5°C	20°C	5°C	20°C
Arla-18	7.95	9.50	9.17	8.12	10.80	8.57	5.60	5.26	5.48	--^d^	16.75	15.58	14.96	14.76	9.61	11.15
ASR-1	8.85	9.87	8.53	8.13	10.44	7.53	5.47	5.18	--	--	15.31	12.46	14.33	11.46	8.97	8.71
ASR-6	8.29	10.23	9.50	8.17	10.45	8.12	5.91	5.35	5.68	--	16.87	14.86	14.95	13.80	9.24	11.16
JFR-1	7.54	9.60	9.52	8.11	10.80	8.93	5.71	5.36	5.57	--	17.27	14.42	15.84	13.23	9.64	11.38
JFR-5	9.42	10.42	10.09	9.02	11.24	9.42	--	--	--	--	15.30	12.65	14.18	11.71	7.92	9.29
OKA-14	8.09	9.26	9.43	8.24	11.23	8.65	5.78	5.30	5.49	--	16.84	14.27	15.42	13.55	8.54	10.41
TSB-8	8.61	9.81	8.84	7.36	9.45	8.80	5.45	5.21	--	--	18.77	13.62	17.34	13.10	8.60	8.49
Yog-3S	8.02	9.96	10.35	8.60	11.62	9.37	5.64	5.47	5.46	--	16.76	14.14	15.12	13.33	8.62	11.07
SEM	0.62	0.62	0.63	0.63	0.58	0.58	0.10	0.10	0.81		0.90	0.90	8.45	8.45	0.87	0.87

^a^ CFS without any treatment.

^b^ CFS with pH neutralized to 6.0.

^c^ CFS with pH neutralized to 6.0 and H_2_O_2_ eliminated.

^d^ No inhibition zone shown.

Inhibition zones (mm) were determined using an agar diffusion assay when target bacteria were used as indicator organisms.

**Figure 1:**
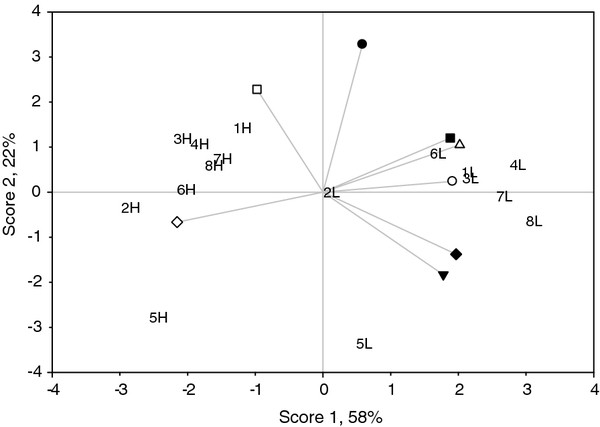
**Principal component analysis (a biplot of antibacterial data of CFS produced by 8 selected bacteriocinogenic LAB cultured at 5 and 20°C).** Indicator organisms included: *Leuc. mesenteroides* CFS, untreated (●), *Leuc. mesenteroides* CFS*,* pH neutralized (○), *B. cereus* CFS, untreated (▼), *L. innocua* CFS, untreated (Δ), *L. innocua* CFS, pH neutralized (■), *L. innocua* CFS, pH neutralized and H_2_O_2_ eliminated (□), *Erw. carotovora* CFS, untreated (♦), and *Ps. fluorescens* CFS, untreated (◊). H-20°C; L-5°C; 1-Arla-18 (*Ent. faecium*); 2-ASR-1 (*Strep. thermophilus*); 3-ASR-6 (*Ent. faecium*); 4-JFR-1 (*Ent. faecium*); 5-JFR-5 (*Lact. casei)*; 6-TSB-8 (*Strep. thermophilus*); 7-OKA-14 (*Ent. faecium*); 8-Yog-3S (*Ent. faecium*).

In conducting a principal components analysis, we intended to figure out that the principal component had as high a variance as possible (accounted for as much of the variability in our data as possible). It was found that score 1 was a contrast between *L. innocua* (control), *L. innocua* (pH neutralized), *Leuc. mesenteroides* (pH neutralized), *Erw. carotovora* (control), *B. cereus* (control) vs *Ps. fluoresecens* (control) (Figure 1). A high score 1 was dominated by the values (inhibitory zones) at 5°C except for *Strep. thermophilus* and *Lact. casei*. All the CFS produced by *Ent. faecium* (Arla-18, ASR-6, JFR-1, OKA-14, and Yog-3S) had a similar antibacterial activity towards all the bacteria tested at both temperatures. The effects of untreated CFS and neutralized CFS from the eight LAB isolates on *L. innocua* highly correlated at 5 and 20°C.

### Action of LAB isolates on three fungi *in vitro*

**Figure 2:**
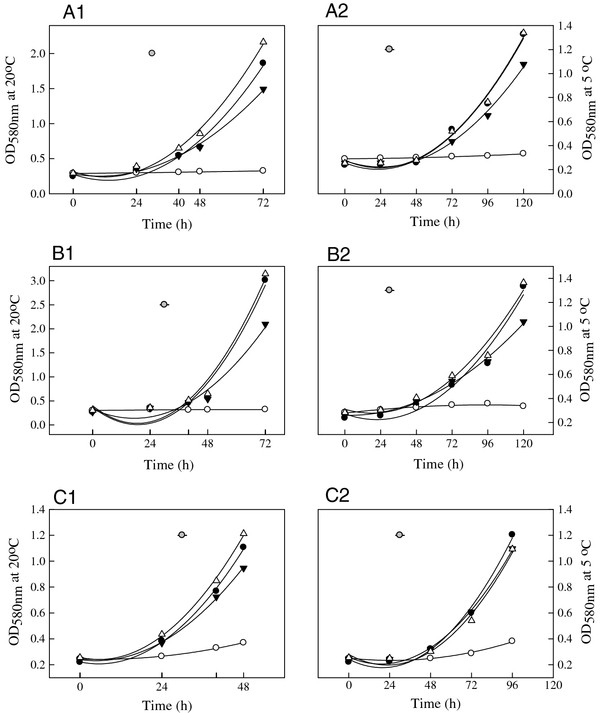
**Influence of BLS-producing LAB on fungal growth (a1, a2 =** ***M. fructicola*at 20 or 5°C*;*b1, b2 =** ***B. cinerea*at 20 or 5°C; c1, c2 =** ***P. expansum*at 20 or 5°C) after incubation at 20°C for 72 h or 5°C for 120 h.** Positive control = Fungal spores grown in MRS broth (●), fungal spores in untreated LAB CFS (○), fungal spores in pH neutralized CFS (▼), fungal spores in CFS after pH neutralization and H_2_O_2_ elimination (Δ). The vertical bar represents standard errors of mean.

No significant difference among the eight LAB isolates was observed with respect to inhibition of spore germination of *M. fructicola, B. cinerea* and *P. expansum* at either 5 or 20°C (*p* > 0.05). Therefore, data for antifungal ability of untreated CFS, pH neutralized CFS, and pH neutralized and H_2_O_2_ eliminated CFS from the eight isolates were averaged, respectively, and compared to the positive controls. At 5 and 20°C, untreated CFS was the most effective at inhibiting fungal growth compared to their controls (Figure 2). Neutralized CFS also had an inhibitory effect on the growth of these fungi (*p* <0.001). However, pH neutralized and H_2_O_2_ eliminated CFS showed no significant effect on fungal growth at 20°C (Figure 2a1, 2b1, and 2c1). Similar results were found at 5°C as the CFS, but not the BLS, produced by the eight LAB were effective in controlling the fungal growth (Figure 2a2, 2b2, and 2c2).

### Effect of selected LAB on microflora naturally occurring on fresh- cut onions

**Figure 3:**
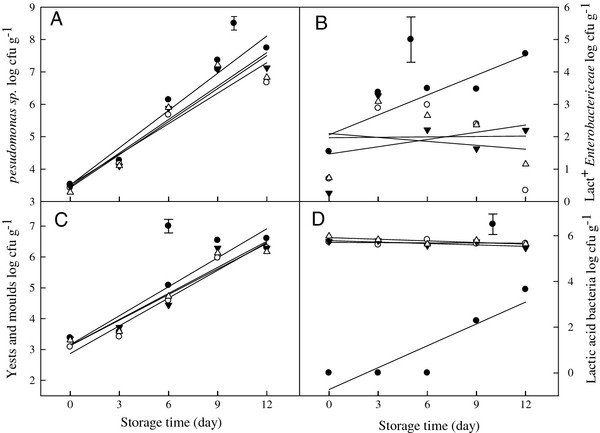
**Influence of BLS-producing LAB on the growth of naturally occurring microorganisms.*** Pseudomonas * sp. (**a**), lactose-positive *Enterobacteriaceae* (**b**), yeasts and moulds (**c**), and LAB viability (**d**) on fresh-cut onion during 12 d storage at 5°C. Fresh- cut onions without LAB inoculation (●), inoculated with *Strep. thermophilu*s (○), inoculated with *Lact. casei* (▼), inoculated with *Ent. facium* (Δ). Fresh-cut onions were inoculated with LAB to a density of 5 × 10^5^ cfu g^-1^. The vertical bar represents standard errors of mean.

Three LAB species inoculated on fresh-cut onions significantly inhibited *Pseudomonas* sp. (*p* = 0.02) (Figure 3a) and lactose-positive (Lac^+^) *Enterobacteriaceae* (*p* = 0.042) (Figure 3b) during 12 d when stored at 5°C compared to non-LAB inoculated controls. On day 12, Lac^+^*Enterobacteriaceae* increased to 4.5 log cfu g^-1^ on control samples while levels of 0.3, 2.2 and 1.1 log cfu g^-1^ were obtained in *Strep. thermophilu*s, *Lact. casei* and *Ent. facium* treated samples, respectively. However, no significant differences were found in yeast and mould levels between LAB inoculated and control samples (Figure 3c). Moreover, no dramatic decrease in the LAB inoculated on the fresh-cut onions was observed over the 12 d storage period (Figure 3d) and no naturally occurring *Listeria* sp. was detected in this study.

### *Listeria* challenge test on fresh-cut onions

**Figure 4:**
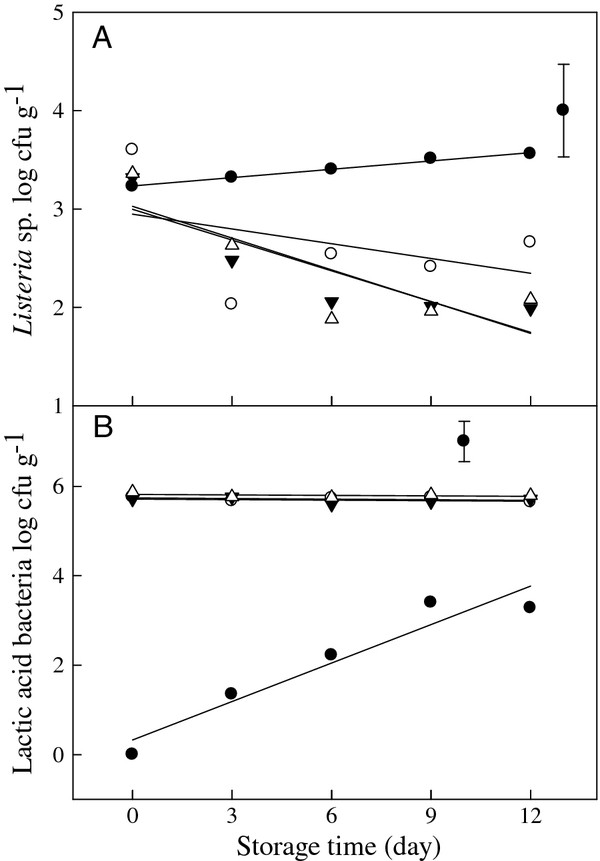
**The viability of*L. innocua*(a) and LAB (b) inoculated onto fresh-cut onions during 12 d storage at 5°C.** Control (in Figure 4a) = fresh-cut onions inoculated with *L. innocua* at 5 × 10^3^ cfu g^-1^ (●); control (in Figure 4b) = LAB naturally occurring on fresh-cut onions (●). Samples inoculated with *Strep. thermophilu*s at 5 × 10^5^ cfu g^-1^ after introduction of *L. innocua* (○), samples inoculated with *Lact. casei* at 5 × 10^5^ cfu g^-1^ after introduction of *L. innocua* (▼), samples inoculated with *Ent. faecium* at 5 × 10^5^ cfu g^-1^ after introduction of *L. innocua* (Δ). The vertical bar represents standard errors of mean.

The initial density of *L. innocua* subsequent to their introduction onto fresh-cut onions was 3.2 log cfu g^-1^ on day 0. During the following 12 d at 5°C, the growth of *L. innocua* was significantly inhibited by the LAB (*p* = 0.042, Figure 4a) as its levels were reduced by 1.0, 1.6, and 1.6 log cfu g^-1^ in samples treated with *Strep. thermophilu*s, *Lact. casei* and *Ent. faecium*, respectively. In contrast, levels of LAB inoculated on the fresh-cut onions remained at the initial inoculation level of 5 × 10^5^ cfu g^-1^ over the 12 d storage at 5°C (Figure 4b).

## Discussion

In the present work we isolated, identified and characterized bacteriocinogenic LAB indigenous to cheese and yogurt and explored their potential as biopreservatives. Two types of media were chosen for the isolation of LAB. First, MRS was used as a medium for LAB which gave a general scope of the flora present in the samples ([Reuter 1985]). M17 agar was also used as a selective medium for the isolation of *Streptococci* sp. Twenty percent of our isolates produced BLS effective against *L. innocua* and 16S rRNA gene sequencing determined that these isolates belonged to four LAB species. ([Sharpe 2009]) reported detecting 8.7% bacteriocinogenic strains among 92 LAB isolated from fresh-cut vegetable products, whereas Sezer and Güven ([2009]) screened 12,700 LAB isolates from milk and meat products and found only 35 exhibited bacteriocin production. Therefore, the choice of food source and media are important for the successfully isolation of bacteriocinogenic LAB.

Using 16S rRNA gene sequencing to identify the 28 bacteriocinogenic LAB isolates, we found that the genus *Enterococcus* was predominant representing 85.7% of the isolates. Another 7.1% were *Streptococcus* sp. whereas the other 7.1% were *Lactobacillus* sp. Furthermore, all the *Enterococcus* sp. were identified as *Ent. faecium*, which was also found to be the most frequently isolated species in cured and semi-cured cheese (Cogan et al. [1997]). López-Díaz et al. ([2000]) found that 40.4% of nearly 500 strains isolated from Valdeón cheese were *Enterococcus* sp.

Bacteriocins can be broken down by some proteolytic enzymes leading to a loss in their antimicobial activity. In our study, we used *L. innocua* or *Lact. sakei* as indicators, and the BLS produced by different LAB stains had various inhibitory effects following treatment with proteolytic enzymes (Table 2). Similar behavior was observed by Khalil et al. ([2009]) with a *Bacillus megaterium*19 strain isolated from a mixture of fermented vegetable wastes. They found that pepsin and trypsin treatment inhibited the bacteriocin activity against *Staphylococcus aureus* more than *Salmonella typhimurium*. Cherif et al. ([2001]) used pepsin, papain, trypsin, chymotrypsin, proteinase K, lysozyme, catalase, DNase and RNase to treat thuricin 7, a bacteriocin produced by *Bacillus thuringiensis* BNG 1.7. They found that the inhibitory activity was only susceptible to proteinase K.

The thermal stability at 80 and 100°C (up to 90 min) (Table 3) of BLS produced by our bacteriocinogenic LAB isolates may constitute an advantage for potential use as biopreservatives in combination with thermal processing in order to preserve food products. However, it should be noted that the antimicrobial effect of these BLS on *L. innocua* and/or *Lact. sakei* was markedly decreased or completely lost after treatment at 121°C for 15 min. [Todorov and Dicks (2009]) reported that bacteriocin ST44AM remained stable at 25, 30, 45, 60 and 100°C for 120 min. However, the activity of this bacteriocin against *L. ivanovii* subsp. *ivanovii* ATCC 19119 was reduced from 3.3 × 10^6^ AU ml^-1^ to 4.1 × 10^5^ AU ml^-1^ after exposure at 121°C for 20 min. Similar results were reported for a bacteriocin produced by *Lactobacillus* CA44 (Joshi et al. [2006]) and also thuricin 7 from *B. thuringiensis* BMG1.7 (Cherif et al. [2001]).

In the present study, eight BLS producing LAB isolates were tested for their antimicrobial effects on three gram-negative, and three gram-positive bacteria, as well as three common spoilage fungi. The results showed that untreated CFS inhibited all test bacteria and fungi except for *E. coli.* However, after pH neutralization and H_2_O_2_ elimination, the CFS inhibited only *L. innocua*. Similar results were reported by [Sharpe (2009]) as they found that the BLS produced by *Lact. lactis* and *Ent. faecium* were able to control *Lact. sakei* and *L. innocua.* However, the strong antimicrobial effects associated with *Lact. lactis* and *Ent. faecium* in our study appeared to be a direct result of the organic acids and the H_2_O_2_ present in the CFS rather than the BLS. In other research, the antimicrobial activity of 12 enterococci strains was confirmed by Hajikhani et al. ([2007]), showing that *Ps. aeruginosa* and *Proteus vulgaris* were sensitive to compounds produced by enterococci but *E. coli* and *Yersinia enterocolitica* were not affected. Trias et al. ([2008a] ) treated apple wounds and lettuce cuts with the LAB strains resulting in reduced counts of *Salmonella typhimurium* and *E. coli* by 1 to 2 log cfu g^-1^, whereas the growth of *L. monocytogenes* was completely inhibited. Cheikhyoussef et al. ([2010]) investigated bifidin I from *Bifidobacterium infantis* BCRC 14602, and reported an increase in bacteriocin activity from 2. 6 × 10^2^ AU mg^-1^ for neutralized CFS to 3.7 × 10^5^ AU mg^-1^ for the purified bacteriocin. Simova et al. ([2009]) achieved a 10^5^ -fold increase in bacteriocin activity after a single peak was assessed upon C2/C18 reversed-phase liquid chromatography purification. The relative low concentration of bacteriocin in the CFS likely contributed to the BLS not being able to inhibit all bacteria and fungi examined in this study. Further investigation is needed to establish the method of direct use of LAB as protective cultures or that of purified bacteriocins on foods.

It has been hypothesized that organic acids act on the cytoplasmic membrane by neutralizing its electrochemical potential and increasing its permeability, thus leading to bacteriostasis and eventual death of susceptible bacteria (Dalié et al. [2010]). The same hypothesis could also explain the susceptibility of some fungal cultures to organic acids (Batish et al. [1997]). However, [Elsanhoty (2008]) suggested that the antifungal effect of LAB could not simply be the result of low pH but is most probably due to the formation and secretion of pH dependent antifungal metabolites. Magnusson and Schnürer ([2001]) found that the antifungal metabolite produced by the *Lact. coryniformis* subsp. *coryniformis* Si3 strain was a small highly heat stable peptide and its activity was stable at pHs between 3.0 and 4.5, but rapidly decreased between 4.5 and 6.0. No inhibitory activity was detected above pH 6.0.

In the present study, selected LAB strains were also inoculated onto fresh-cut onions to investigate *in vivo* application of these isolates. These isolates significantly inhibited the growth of *Pseudomonas* sp. and Lac^+^*Enterobacteriaceae* during storage at 5°C (Figure 3a, 3b). Similar results were reported by [Sharpe (2009]); when *L. lactis* and *Ent. faecium* were inoculated onto fresh-cut salads, the growth of *Pseudomonas* sp., yeasts and total coliforms were remarkably reduced. Vescovo et al. ([1995,1996]) reported that the inoculation of ready-to-use vegetables with selected strains of LAB effectively controlled the growth of undesirable bacteria. In our challenge tests, *L. innocua* inoculated onto fresh-cut onions was reduced by 1 to 1.6 log cfu g^-1^ after 12 d storage at 5°C due to the presence of selected LAB strains. Although the inoculated LAB loads did not increase during the storage period, they still significantly inhibited the growth of *L. innocua*. Further investigations into the mechanisms of inhibition and determination of the optimal growth conditions for LAB to produce BLS are necessary. Moreover, more studies are required to envisage the effectiveness of LAB and BLS on other food products.

## Competing interest

The authors declare that they have no competing interests. Contribution no. 2379 of the Atlantic Food and Horticulture Research Centre, Agriculture and Agri-Food Canada (AAFC).
